# Structural and functional analysis of SMO-1, the SUMO homolog in *Caenorhabditis elegans*

**DOI:** 10.1371/journal.pone.0186622

**Published:** 2017-10-18

**Authors:** Parag Surana, Chandrakala M. Gowda, Vasvi Tripathi, Limor Broday, Ranabir Das

**Affiliations:** 1 National Centre for Biological Sciences, Tata Institute of Fundamental Research, Bengaluru, India; 2 Department of Cell and Developmental Biology, Sackler Faculty of Medicine, Tel Aviv University, Tel Aviv, Israel; George Washington University, UNITED STATES

## Abstract

SUMO proteins are important post-translational modifiers involved in multiple cellular pathways in eukaryotes, especially during the different developmental stages in multicellular organisms. The nematode *C*. *elegans* is a well known model system for studying metazoan development and has a single SUMO homolog, SMO-1. Interestingly, SMO-1 modification is linked to embryogenesis and development in the nematode. However, high-resolution information about SMO-1 and the mechanism of its conjugation is lacking. In this work, we report the high-resolution three dimensional structure of SMO-1 solved by NMR spectroscopy. SMO-1 has flexible N-terminal and C-terminal tails on either side of a rigid beta-grasp folded core. While the sequence of SMO-1 is more similar to SUMO1, the electrostatic surface features of SMO-1 resemble more with SUMO2/3. SMO-1 can bind to typical SUMO Interacting Motifs (SIMs). SMO-1 can also conjugate to a typical SUMOylation consensus site as well as to its natural substrate HMR-1. Poly-SMO-1 chains were observed *in-vitro* even though SMO-1 lacks any consensus SUMOylation site. Typical deSUMOylation enzymes like Senp2 can cleave the poly-SMO-1 chains. Despite being a single gene, the SMO-1 structure allows it to function in a large repertoire of signaling pathways involving SUMO in *C*. *elegans*. Structural and functional features of SMO-1 studies described here will be useful to understand its role in development.

## Introduction

**S**mall **U**biquitin like **Mo**difier (SUMO) is a post translational modifier involved in multiple eukaryotic pathways[1]. SUMO conjugation or SUMOylation presents a SUMO tag on the target protein. The SUMO tag generates a new functional interaction surface comprising the conjugated SUMO and a SUMO-Interacting Motif in the target protein[2]. In some cases, SUMOylation can obstruct interaction surface of the target resulting in, for example, disruption of binding to chromatin–as observed for transcription factor SP3[3], p53[4]—or in alteration of subcellular localization of proteins like PML[5]. SUMOylation is also known to affect other interaction surfaces and conformations of the target protein[6,7]. The reversible nature of SUMO modification allows it to regulate and take part in many cellular processes like transcription, nuclear transport, chromatin remodelling, DNA repair, mitosis, cell cycle progression, differentiation etc. across eukaryotes[1]. Consequently SUMOylation is a prime area for disease research and intervention[8,9], especiallycancer[10], cardiomyopathies[11,12] and pathogenic diseases[13]. Every eukaryote, studied so far, from protozoans to metazoans, including plant and fungi, expresses at least one SUMO like protein or SUMO precursor protein[14].

SUMO is an Ubiquitin like modifier and is conjugated to target proteins in a manner similar to ubiquitination. It is charged by E1 enzyme (heterodimer of Sae1 or Aos1 and Sae2 or Uba2) using ATP hydrolysis. The C-terminal GG motif of SUMO is transferred to E2 enzyme (Ubc9) by thioester linkage. Ubc9 protein can transfer SUMO to a target protein’s primary amine[15].However, E3 SUMO ligases such as PIAS[16] and RanBP2[17] accelerate the SUMO conjugation. The consensus site for SUMO modification is ψKXE on the target protein where ψ is a bulky hydrophobic residue such as L/I/V. The consensus site is also found in some SUMO paralogs raising the possibility of poly-SUMO chains. Paralogs lacking the consensus site have also been recently shown to form poly-SUMOylated chains[17]- the mechanism of which is not known. De-conjugation is carried out by SUMO-specific proteases such as ULP/SENP[18]. SUMO proteases also carry out maturation of SUMO to expose GG motif at the C-terminal tail. Most of the interactions of SUMO tag with downstream effectors are mediated through a SUMO Interaction Motif (SIM) on the effector. The motif consists of branched hydrophobic residues with serine and/or acidic residues on either side. The SIM forms a beta strand and sits in the groove between beta strand 2 and alpha helix of SUMO.

Mammals usually express more than one paralog of SUMO. Humans express at least 5 –SUMO1 to SUMO5. While there is some functional redundancy in some paralogs[19] they have specific functions limited to specific time and space of expression. Paralog specific conjugation of targets and their deconjugation has been observed[20,21]. There are also differences in SIM affinities and preferences. These differences can be explained on the basis of different structural and sequence features. SUMO1 of humans is quite different from other human SUMO paralogs and forms a separate phylogenetic clade. Invertebrates like Yeast (*Saccharomyces cerevisiae*)[22], nematode (*C*. *elegans*)[23] and fruitfly (*Drosophila melanogaster*)[24] express a single SUMO gene. Although multiple protein targets have been identified for SUMO conjugation it is not clear how the single SUMO protein is utilized and regulated for diverse cellular pathways and developmental stages in these organisms.

The nematode *Caenorhabditis elegans* is an important model organism for studying multiple aspects of metazoan developmental and cell biology[25]. The developmental changes and the lineage of each cell in the adult nematode has been mapped out. Many aspects of the developmental and cellular pathways in *C*. *elegans* are conserved in mammals. *C*. *elegans* has only one SUMO ortholog, SMO-1, which is essential for nematode development[23,26]. RNAi against SMO-1 causes embryonic arrest[26]. Homozygous deletion mutants of SMO-1 are sterile and develop abnormal gonad, germ line cells and vulva[27]. SMO-1 is widely expressed in different tissues and development stage of the nematode–more than 200 target proteins were identified for SMO-1 modification in a mass spectrometry based proteomics study[28]. Apart from SMO-1 deletion mutation, impairment of SMO-1 conjugation and/or de-conjugation (by ULP proteins) has been shown to result in embryonic abnormalities, improper muscle development, cell cycle progression and epidermal morphogenesis[29,30]. The *C*. *elegans* model thus provides an opportunity to study how SUMO modifications affect developmental and morphogenetic events.

Here we study the unique features, functions and modifications of SMO-1. We determine the high resolution solution structure of SMO-1. The N-terminal region of SMO-1 is found to be highly dynamic. Although the SMO-1 sequence is similar to SUMO1, its surface electrostatic profile is closer to SUMO2. SMO-1 binds to typical SIMs and conjugates to typical SUMOylation sites. Moreover, SMO-1 can form poly-SMO-1 chains in *in-vitro* conditions. DeSUMOylation enzymes like SENP2 can depolymerize poly-SMO-1 chains as well as cleave immature SMO-1. Overall, SMO-1 was found to integrate features of the multiple human SUMOs. Despite being a single gene, the SMO-1 structure allows it to function in the large repertoire of signaling pathways involving SUMO in *C*. *elegans*.

## Methods and materials

### Cloning and protein purification

SMO-1 was cloned into pET15b vector from a synthetic DNA using BamHI and NdeI restriction enzymes to cut at the two ends of the SMO-1 construct. DNA sequence of all constructs were confirmed by sequencing. The sequenced clone was transformed in BL21(DE3) bacterial cells and plated. A single colony was grown in 10ml Luria Bertani (LB) broth for 6hr. Cells were pelleted and transferred in 50ml M9 minimal media having ^13^C labelled Glucose and ^15^N labelled Ammonium Chloride. After overnight incubation at 37°C, this culture was used to inoculate 1litre of same M9 minimal media. The culture was induced at O.D. of 0.8 with 0.5mM IPTG and further incubated at 37°C for 3–4 hours. Cells were pelleted and resuspended in 50mM Tris-Cl pH 8.0 (at 4°C), 250mM NaCl, 5% v/v Glycerol, 5mM beta-mercaptoethanol, 1mM phenyl methyl sulfonyl fluoride and 0.01% v/v TritonX100. Sonication at 50% amplitude for 20 cycles of 5sec on and 25sec off lysed the bacterial cells. The sample was then centrifuged at 17000 rpm to separate soluble proteins from cell debris. Supernatant thus collected was filtered and incubated with Ni-NTA column (GE Healthcare). Column was washed with 20mM, 50mM, 100mM, 250mM and 500mM Imidazole in buffer having 50mM Tris-Cl pH 8.0 (at 4°C), 250mM NaCl, 5% v/v Glycerol, 5mM beta-mercaptoethanol and 0.01% v/v TritonX100. Fractions containing SMO-1 protein were pooled and was further purified by Size Exclusion Chromatography (SEC) on Superdex 75 column (GE Healthcare). SEC was run with buffer 25mM Tris-Cl pH 8.0 (at 4°C), 250mM NaCl and 2% v/v Glycerol. For NMR experiments, protein containing fractions were buffer exchanged to Sodium Phosphate pH 6.0 buffer with 150mM NaCl. For storage in -20°C, 0.03% w/v Sodium Azide, 1x Protease Inhibitor Cocktail (Roche) and 10% v/v Glycerol was maintained in the concentrated protein samples. Unlabelled SMO-1 protein was purified and processed similarly but with the unlabelled Glucose and the unlabelled Ammonium Chloride in the bacterial growth media. SMO-1 was subcloned into pET15b vector using BamHI and NdeI restriction enzymes to cut at the two ends of the SMO-1 construct. SMO-1 construct was also cloned upstream of Yellow Fluorescent Protein (YFP) in pET15b via a flexible linker coding for GGSGG. DNA sequence of all constructs were confirmed by sequencing.

HMR-1 C-tail constructs[29] were expressed in bacteria and purified using standard protocols of Ni-NTA affinity purification followed by Size Exclusion Chromatography. Other proteins, human Aos1/Uba2, Ubc9 and SUMO1 were also purified similarly.

## Circular dichroism measurements

Far UV CD measurements were carried out on Jasco J-815 spectropolarimeter. Protein samples of concentration 7.5 μM were prepared in 25mM Tris-Cl pH 7.5 @ 25°C. Measurements were taken at 25°C in 0.1cm path length cuvette. Fifteen scans were averaged and appropriate buffer scans were subtracted from scans of protein samples. Mean Residual Ellipticity was plotted against wavelength.

### Structure determination by NMR

^13^C, ^15^N-labeled SMO-1 protein in 25mM Sodium Phosphate pH 6.0 buffer with 150mM NaCl was concentrated up to 1mM, dissolved in 90%-10% v/v H_2_O-D_2_O buffer. NMR spectra were recorded at 298K on 800 MHz Bruker Avance III HD spectrometer equipped with a cryo-probe head. BEST[31] versions of 3D HN(CO)CACB, HNCACB and HNCO, and 2D ^1^H-^15^N-HSQC experiments were used for backbone assignments. Side chain assignments were determined by recording 3D H(CCO)NH, (H)CC(CO)NH and HCCH-TOCSY experiments. 2D (HB)CB(CGCD)HD experiment data along with 2D ^13^C aromatic HSQC spectra was also used for side chain assignment. Standard 3D ^15^N-NOESY-HSQC and ^13^C-NOESY-HSQC experiments with a mixing time of 150ms and 100ms respectively were used for obtaining distance restraints. All NMR data were processed in Bruker Topspin3.5pI5 and analysed by NMRFAM-SPARKY 1.3[32] software. Following peak-picking of the backbone and side chain experimental data in SPARKY, the peaks were assigned manually. Automatic peak picking was performed for NOESY spectra using NMRFAM-SPARKY 1.3[32]. NOESY peak assignments were performed in CYANA[33] and corrected over multiple cycles of structure building. The backbone assignments were 98% complete and the side chain assignments were 74% complete.

TALOS+[34] was used to predict the phi and psi torsion angles from the assigned chemical shifts of backbone atoms. Phi/psi torsion angles and NOESY based distance restraints were used to determine the solution structure of SMO-1 by CYANA software[33]. 400 starting structures were calculated and out of which 20 lowest energy structure models were chosen to represent the final structural ensemble. Explicit water refinement was performed using Ponderasa web server[35]. The backbone dihedral angles of the final converged structures were evaluated by the Molprobity[36] and PSVS[37] suite of programs. Details of the restraints and structure evaluation are provided in Table 1.

**Table 1:** NMR and refinement statistics of SMO-1.

**Distance Restraints (NOE)**	
Short (|i-j|< = 1)	814
Medium range (1<|i-j|<5)	270
Long (|i-j|> = 5)	383
Total	1467
**Dihedral Restraints**	
Phi	66
Psi	66
**RMS Deviation from Ideal geometry**	
Bond angles (°)	0.2
Bond lengths (Å)	0.001
**RMSD (Å)**^**a**^	
All backbone	0.42 (±0.17)(0.25..0.89; best 20 structures)
All heavy atoms	0.88 (±0.17)(0.72..1.29; best 20 structures)
**Ramachandran Plot**^**a**^	
Most Favoured regions (%)	89.6
Allowed regions (%)	9.7
Disallowed regions (%)	0.8

^a^ Calculated using amino acid residues 11–88 of 20 lowest energy structures of SMO-1 deposited in PDB.

### NMR relaxation experiments

^13^C, ^15^N-labeled SMO-1 protein was kept in 25mM Sodium Phosphate pH 6.0 buffer with 150mM NaCl. All NMR samples had 10% v/v D_2_O. ^15^N spin relaxation experiments carried out were longitudinal (T1), transverse (T2) time constants and ^15^N {^1^H} steady-state Nuclear Overhauser Enhancement (hetNOE). These experiments were performed at 298K.The spectra for T1 were acquired with T1 relaxation delays varying from 0.002 sec to 0.4 sec (0.002, 0.005, 0.01, 0.03, 0.05, 0.1, 0.2 0.4) and the spectra for T2 were acquired with T2 relaxation delay varying from 0.002 sec to 0.2 sec (0.002, 0.008, 0.016, 0.024, 0.032, 0.064, 0.096, 0.2). The T1 and T2 values are determined by single exponential fit of the decay of the peak intensities. The heteronuclear steady-state ^15^N-^1^H NOE values are obtained by the ratio of the cross peak intensities in the saturated spectrum to those in the unsaturated spectrum. All NMR relaxation experiment data were processed by Bruker Topspin3.5pI5 and analysed by NMRFAM-SPARKY 1.3[32] software.

### NMR binding experiments

To study SMO-1-SIM interaction by NMR, both ^13^C, ^15^N-labelled SMO-1 and unlabelled SIM peptides (PML-SIM, sequence—WGEAEERVVVISSSEDSDAEN and IE2-SIM, sequence–DTAGCIVISDSEEEQGEEW) were dialyzed in 25mM Sodium Phosphate pH 6.0 buffer with 150mM NaCl. ^15^N-HSQC of SMO-1 was recorded at 298K. Peptide was added to this SMO-1 NMR sample up to a molar ratio of 1:5 (SMO-1:SIM). ^15^N-HSQC was recorded at each titration point. The chemical shift perturbations (CSP) between the free and the bound form were calculated as CSP = [(δ^H^_free_− δ^H^_bound_)^2^+ ((δ^N^_free_− δ^N^_bound_)/5)^2^]^1/2^, where δ^H^ and δ^N^ are the chemical shift of the amide hydrogen and nitrogen, respectively. Chemical shift of SMO-1 peaks were analysed to obtain dissociation constants (K_d_). The titration data was fit in 1:1 protein:ligand model using the equation CSP_obs_ = CSP_max_ {([P]_t_+[L]_t_+K_d_)—[([P]_t_+[L]_t_+K_d_)^2^–4[P]_t_[L]_t_]^1/2^}/2[P]_t_, where [P]_t_ and [L]_t_ are total concentrations of protein and ligand at any titration point.

### Accession numbers

The coordinates of NMR based solution structure of SMO-1 is deposited in Protein Data Bank with the accession number: **5XQM**. The NMR chemical shifts have been submitted to BMRB database under the accession number: **36096**.

### *In vitro* SMO-1 conjugation assays

A synthetic peptide, sequence–PLIKQEDIKPEPDFTIQYRNKIIDTAGCIVISDSEEE with Fluorescein iso-thiocyanate (FITC) label at the N-terminus was used as substrate. 10mM of peptide was incubated with 50 μM human SUMO1 or *C*. *elegans* SMO-1 in presence of 1 μM E1 enzyme (human Aos1/Uba2) and 2.5 μM E2 enzyme (human Ubc9). Reaction was carried out at 4°C for 12 hours. The reaction was started by adding 1 mM ATP. Reaction buffer contained 20 mM Tris-Cl pH 8.5, 250 mM NaCl, 5 mM MgCl_2_, 0.1% v/v Tween 20. For reactions involving HMR-1A C-tail protein and the HMR-1A-K2R mutant protein, 5μM of HMR-1A C-tail protein (or HMR-1A-K2R mutant) and 5μM of human SMO-1 was used. FITC was conjugated to primary amine of SMO-1 when HMR-1A C-tail protein was used. Reaction buffer was the same as above. The reaction was carried out at 37°C for 4 hours. All reactions were stopped by adding reducing SDS loading dye. Reaction samples were run on 12% w/v SDS PAGE and analysed by probing FITC fluorescence at 519 nm wavelength (excitation wavelength– 495 nm).

### *In vitro* SMO-1 deconjugation assays

Deconjugation Assays were performed by adding 10μM SENP2 to poly-SUMO1/2 and poly-SMO-1 chains in the same reaction mix used for conjugation. Deconjugation reaction was carried out at 25°C for 2–3 hours. Reactions were stopped by adding reducing SDS loading dye. Reaction samples were run on 12% w/v SDS PAGE and analysed by probing FITC fluorescence at 519 nm wavelength (excitation wavelength– 495 nm). SMO-1-YFP was checked for deconjugation in 50mM Tris-Cl pH 7.5 @ 25°C, 150mM NaCl, 1mM DTT reaction buffer. SENP2 was mixed with SMO-1-YFP and incubated at temperatures from 25–37°C for 6 hours. The reaction mix were then run separately on two SDS-PAGE gels for imaging using fluorescence and for staining with Coomassie Brilliant Blue R-250 Dye.

### Sequence and structure analysis

Protein sequence alignment was done by Clustal Omega software. Structural alignments and all images of structures were prepared using Chimera[38]. The structure was first prepared by PDB2PQR webserver[39] and then APBS[40] tool was used in Chimera to perform electrostatic calculations.

## Results

### Solution structure of SMO-1

**Fig 1:**
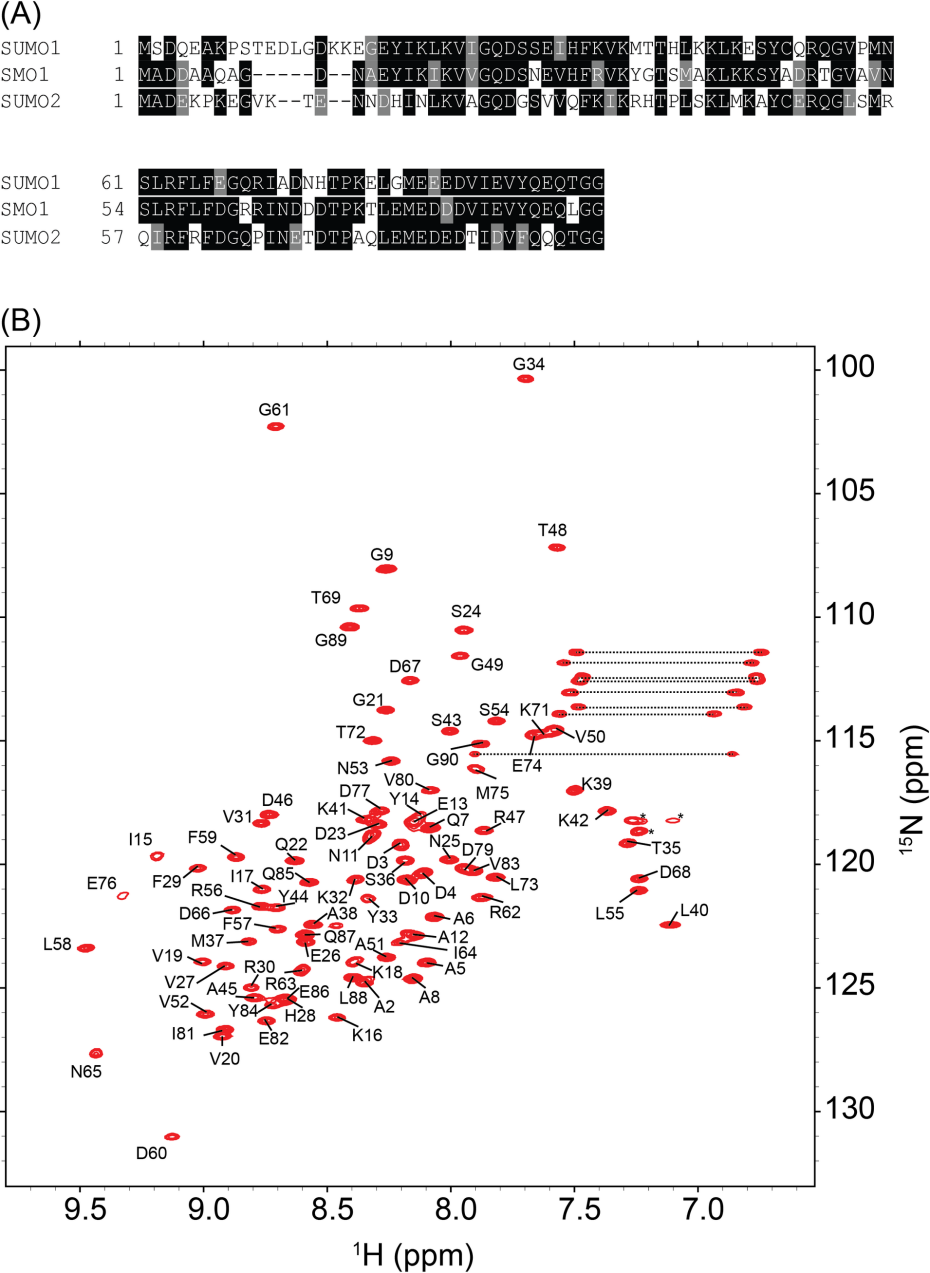
Sequence and structural examination of SMO-1. (A) Multiple sequence alignment of SMO-1 with its homologues SUMO1 and SUMO2. Identical residues across the homologues are boxed in black while similar residues are boxed in grey. (B) ^15^N-HSQC of SMO-1 protein is shown with backbone amide peaks labelled by residue numbers. The side chain amides are connected by dashed lines. The folded peaks of arginine side chains are marked by an asterisk.

Sequence alignments show that SMO-1 has higher sequence similarity to human SUMO1 than to SUMO2/3 (Fig 1A). A ^15^N-edited HSQC NMR spectrum was collected of the purified ^15^N, ^13^C-SMO-1. The amide peaks in the spectra were well-dispersed, indicating a folded protein (Fig 1B). Chemical shift data were obtained using a series of 3D and 2D NMR experiments as discussed in the Methods and Materials section. Dihedral restraints were obtained from chemical shift analysis by TALOS+. Proton-proton NOE restraints were obtained from^15^N-edited NOESY-HSQC and ^13^C-edited NOESY-HSQC experiments. Finally, taking the predicted torsion angles and the distance restraints from NOEs, a structure calculation of SMO-1 was performed. The final structures of SMO-1 converged with good statistics and a low RMSD (0.5 Å, 78 Ca pairs) at the core of the protein (Table 1).

**Fig 2:**
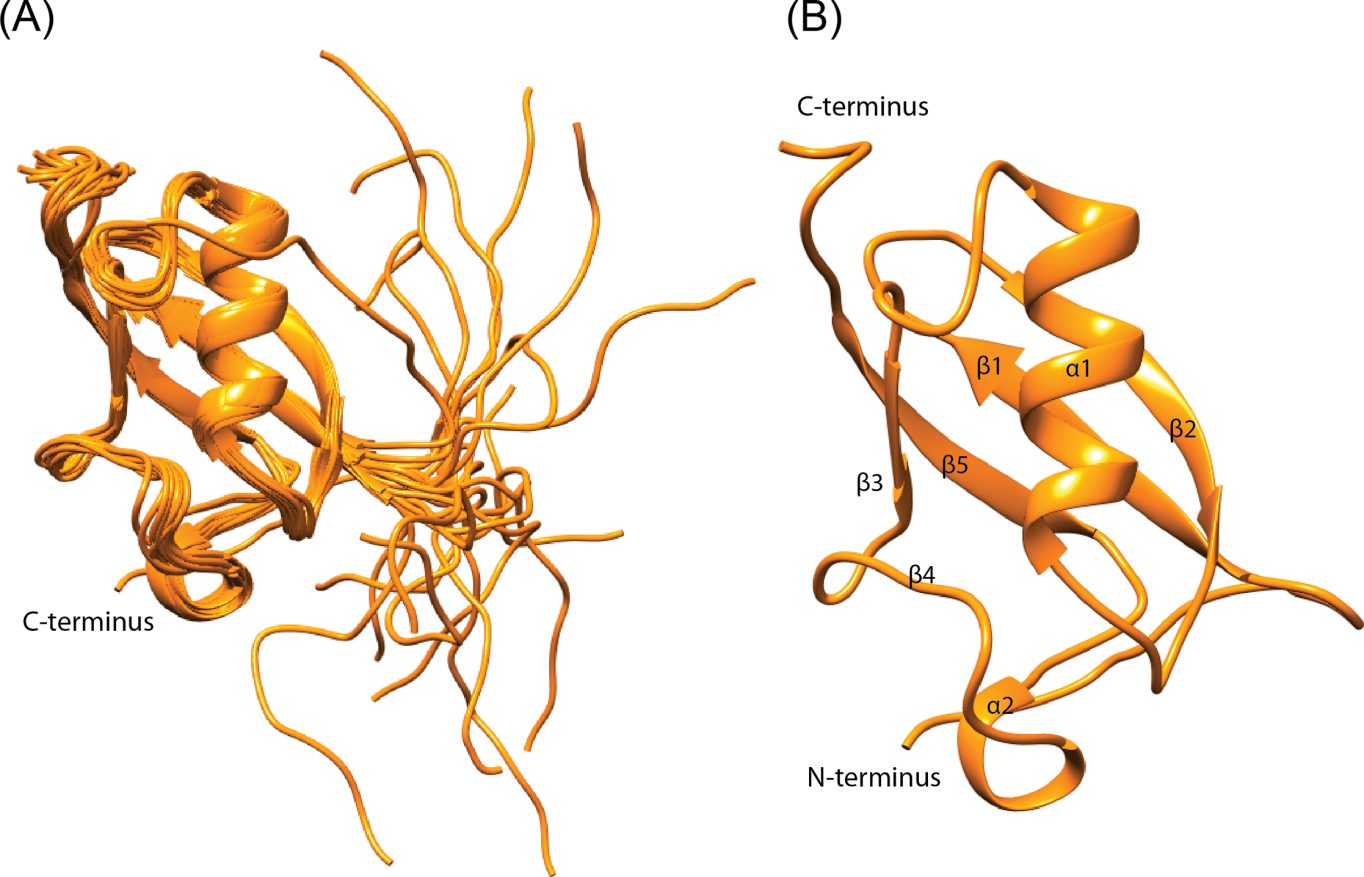
NMR solution structure of SMO-1. (A) 20 lowest energy NMR solution structures of SMO-1calculated by CYANA. The SMO-1 chains (in orange) are depicted in cartoon representation. (B) The best model structure of SMO-1 protein is shown in orange. The chain termini and secondary elements are labelled.

**Fig 3:**
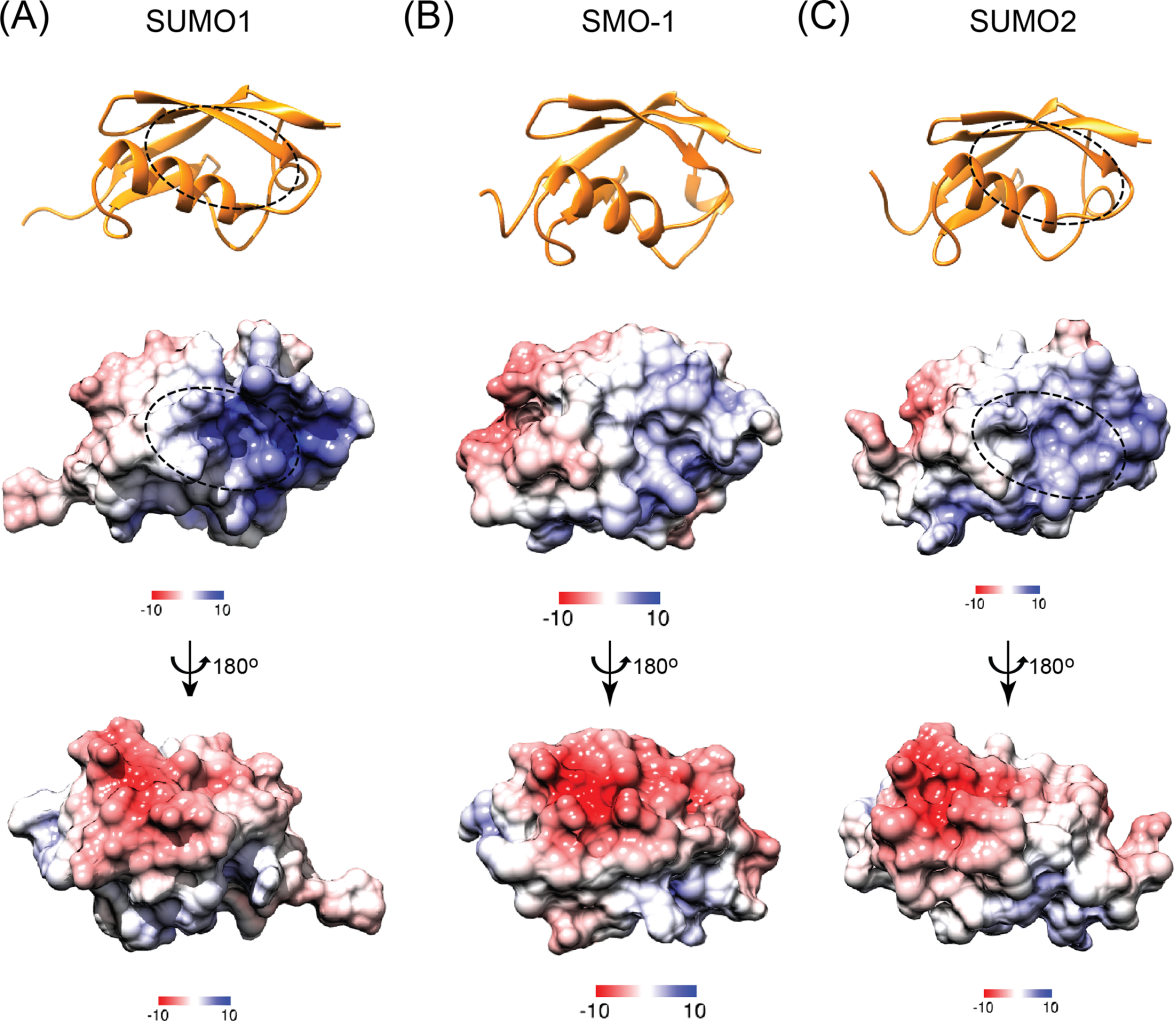
Comparison of structures and electrostatic surface potential between SMO-1 and its SUMO homologues. (A), (B) and (C) The top panel shows the cartoon representation of SUMO1, SMO-1 and SUMO2 (all in orange). The SIM interacting region of SUMO1 and SUMO2 is marked by an oval in broken line. The middle panel shows the APBS calculated (at 298 K) electrostatic surface potential of respective proteins in the orientation shown in top panel. The bottom panel shows the same electrostatic surface potential at an orientation rotated 180 degree about the vertical shown as shown. Color gradient scheme of the electrostatic surface is shown for each surface. Positively charged surfaces are colored blue, neutral surfaces are in white and negatively charged surfaces are in red. The unit of the color gradient shown is kcal/(mol.*e*).

The NMR derived solution structure ensemble shows a well folded beta-grasp core in SMO-1 (Fig 2A). The beta-grasp fold includes five beta-strands (β1-β5) and a single helix (α1). A long loop with a small alpha helical turn is present between the β4 and β5 strands. The SMO-1 structure superimposes well with an RMSD of 2.1Å to SUMO1 and an RMSD of 2.5Å to SUMO2 over 78 Cα pairs at the core of the protein, indicating that the fold is conserved in *C*. *elegans*. The 14 residue long N-terminal tail is the most flexible region followed by the C-terminal harbouring the GG motif (Fig 2B). The surface of SMO-1 shows a negatively charged face and a positively charged face (Fig 3). The major protein interaction interface lies at the groove between the alpha helix 1 and the beta strand 2, where SIM containing peptides dock. The groove is positively charged in SUMO1 while in SMO-1 (and in SUMO2/3) the positive charge is significantly less. This may affect binding to phosphorylated SIM and/or SIM peptides containing acidic residues. The groove on the surface opposite to SIM binding area is used by dipeptidyl peptidase 9 to specifically interact with SUMO1 over other SUMO paralogs[41]. The specificity hinges on the presence of Histidine in the loop starting after beta3 in SUMO1. This creates a positively charged surface which is continuous to the SIM binding site. SMO-1 although more similar to SUMO1 has an Aspartate, like that in SUMO2/3, instead of Histidine (S1 Fig). SMO-1 (and SUMO2/3) has a neutral or slightly negative charge at this part. This loop also enables specific interaction between SUMO2/3 and SUMO proteases such as SENP6 and SENP7[42]. Aside from these two surface features SMO-1 is more similar to SUMO1 than to SUMO2/3. This includes the beta strands, the alpha helix and the C-terminal tail. This suggests that SMO-1 has a core packing similar to SUMO1. In addition, the surfaces and residues in SMO-1 towards E1enzyme, E2enzyme and SUMO protease interaction are more similar to those in SUMO1 than to those in SUMO2/3[43].

### Dynamics of SMO-1

**Fig 4:**
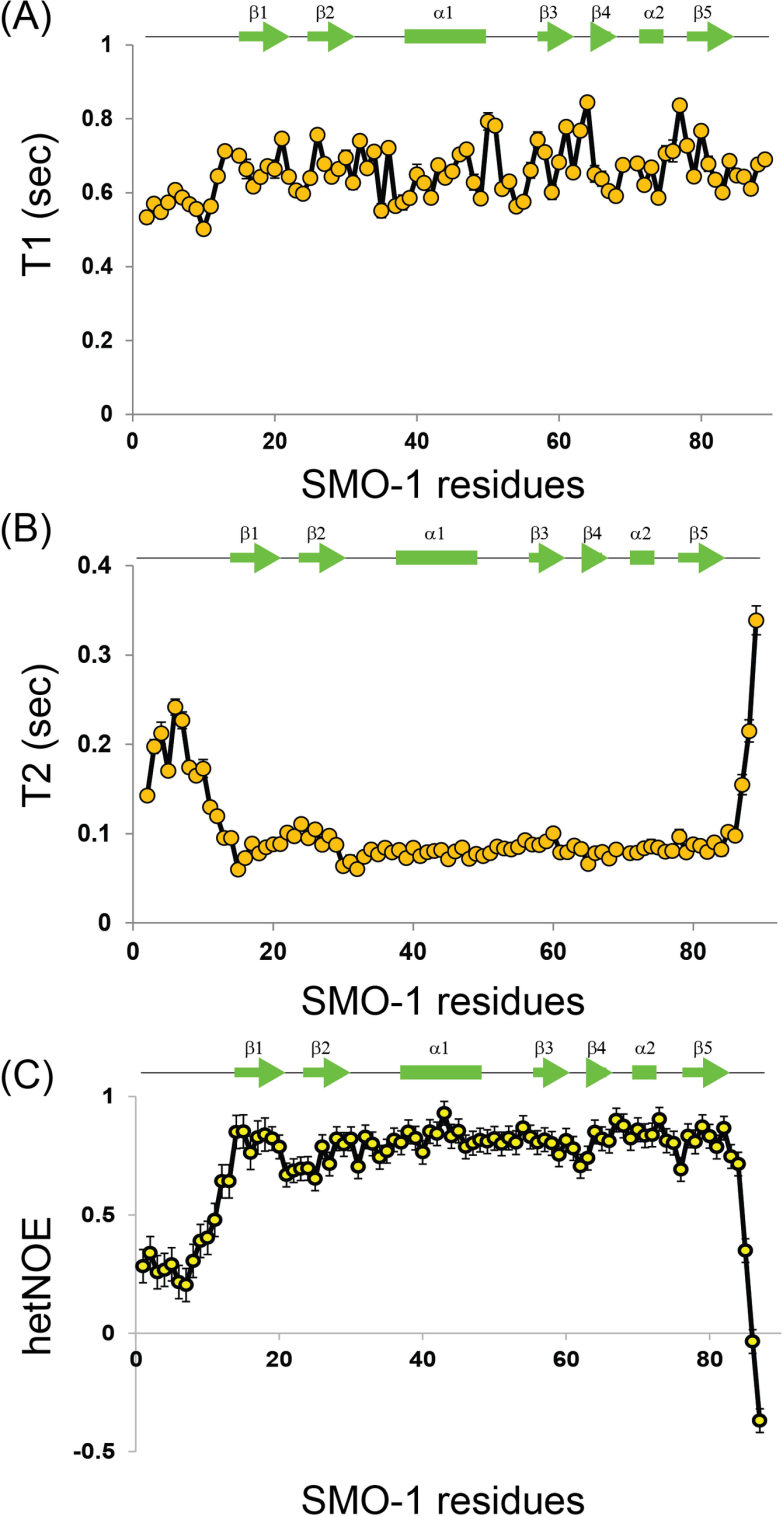
Backbone dynamics in SMO-1 protein at picosecond-nanosecond timescale. ^15^N longitudinal relaxation times constants T1 (A), ^15^N transverse relaxation time constants T2 (B) and {^1^H}-^15^N hetNOEs values (C) of backbone amides for all observable residues in SMO-1. Lower T1 values, higher T2 values and hetNOEs closer to zero or less than zero indicated higher ps-ns backbone dynamics. The secondary structure elements corresponding to SMO-1 residues are shown in green line above each plot. The standard error bars of the fitted parameters values are shown for each plot.

We probed the picosecond-to-nanosecond dynamics of individual residues of SMO-1 protein by spin relaxation NMR experiments[44–46]:^15^N longitudinal spin time constant (T1), transverse spin time constant (T2) and ^1^H-^15^N steady-state heteronuclear Overhauser enhancements (het-NOEs).LowT1, highT2, and low or negative hetNOE values indicates more dynamics in picosecond to nanosecond timescales. The N-terminal and the C-terminal of SMO-1 are highly flexible–as evidenced from the relaxation parameters and the negative hetNOE values (Fig 4). The protein core including the beta strands and the helices is on an average more rigid and hence structured.

### SMO-1 can bind typical SUMO Interaction Motifs

**Fig 5:**
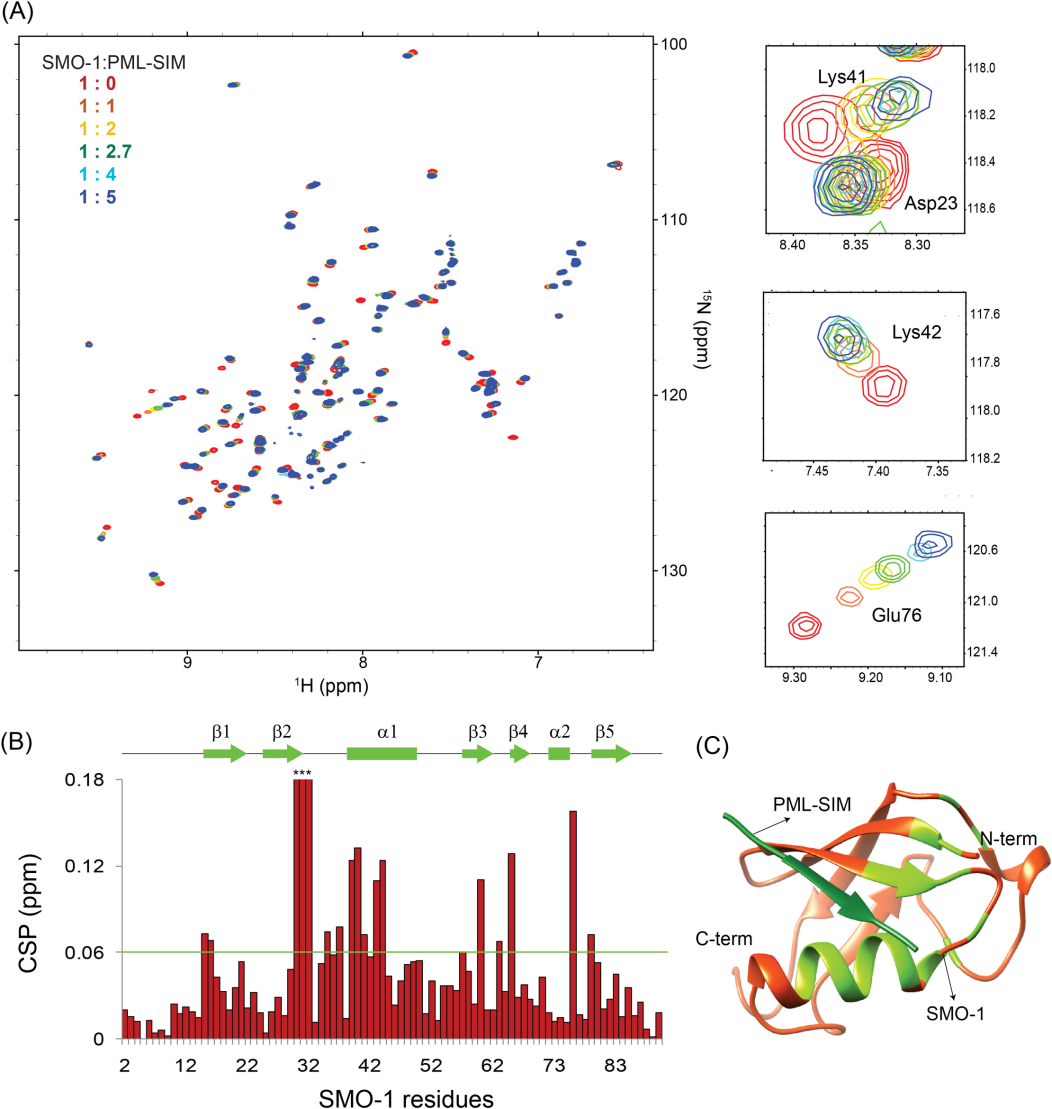
SMO-1 binds SIM with micromolar affinity. (A) Overlay of ^15^N-HSQC spectra of SMO-1 protein with increasing amounts of unlabelled PML-SIM peptide. Peaks are colored according to different ratios of SMO-1:PML-SIM to highlight chemical shifts. On the right are three panels showing zoomed in views focusing on chemical shifts of specific residues. The residues are indicated. (B) Chemical shift perturbation (CSP) of ^15^N-HSQC peaks between the 1:5 SMO-1:PML-SIM sample and the 1:0 SMO-1:PML-SIM sample. The chemical shift perturbations (CSP) between the free and the bound form were calculated as CSP = [(δ^H^_free_− δ^H^_bound_)^2^+ ((δ^N^_free_− δ^N^_bound_)/5)^2^]^1/2^, where δ^H^ and δ^N^ are the chemical shift of the amide hydrogen and nitrogen, respectively. The green horizontal line indicates twice the standard deviation above average. Some of the amide peaks of SMO-1 exchange out upon titration and don’t show up in the HSQC spectra. These amide residues are marked with asterisk and high CSP in the figure. These high CSPs were omitted in the calculation of average and the standard deviation. The secondary structure elements corresponding to SMO-1 residues are shown in green line above the plot. (C) Modelled structure of SMO-1 (orange) bound to PML-SIM (dark green). SMO-1 residues which showed CSP higher than twice the standard deviation above average CSP is coloured green. The N-terminal 12 residues are not shown in the structure for clarity.

SUMO modification can alter the function or localization of the protein by introducing an epitope that can now bind to receptors that have SUMO Interacting Motifs or SIMs. Hence, interaction with SIMs is an important property of the SUMO proteins. To assess if SMO-1 can possibly function by such mechanism we tested the binding of SMO-1 with a couple of typical SIMs. The SIM from Promyelocytic leukemia protein (PML) was titrated in a sample of ^15^N-labeled SMO-1 and the binding was monitored using ^15^N-edited HSQCs (Fig 5A). SMO-1 peaks shifted consistently upon titration of PML-SIM, indicating binding of SMO-1 with the SIM. The peak-shifts were fit against the ligand:protein concentration to determine the K_d_ between SMO-1 and PML-SIM to be ~31(±6) μM(S2 Fig), consistent with typical K_d_ observed in SUMO/SIM interactions. We also tested the binding of a SIM from the IE2 protein in human cytomegalovirus with SMO-1. Again, consistent peak-shifts were observed in the SMO-1 HSQCs (S3 Fig). The dissociation constant K_d_ between SMO-1 and IE2-SIM was calculated to be 108(±17) μM. For both IE2-SIM and PML-SIM, the majority of significant chemical shift perturbations (CSP) was observed in the region between β2 and α1 of SMO-1, which is the canonical binding site of SIMs (Fig 5B and 5C and S3 Fig).

### SMO-1 forms chains in-vitro

**Fig 6:**
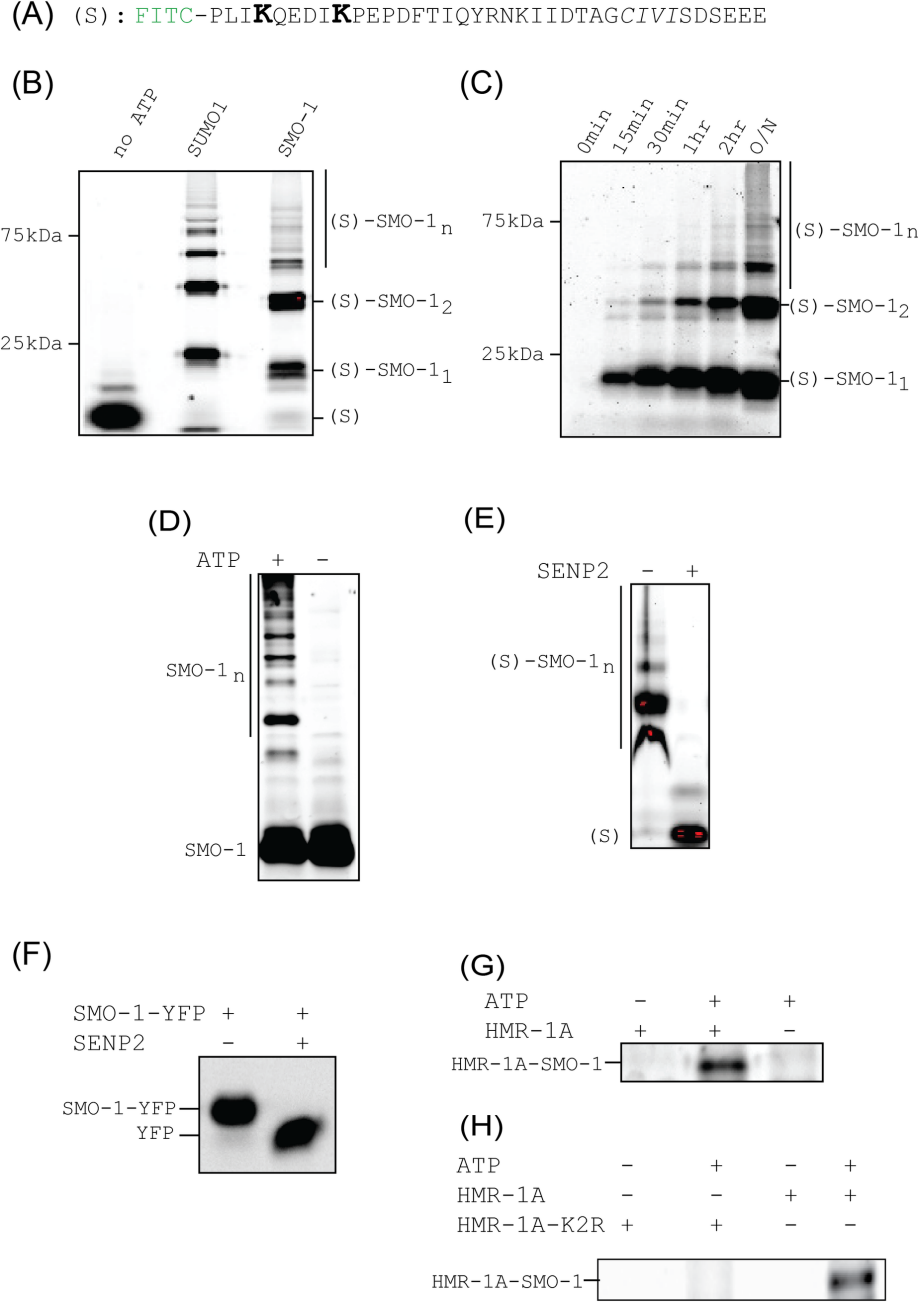
SMO-1 forms chains in *in vitro* SUMOylation assays. (A) Sequence of FITC fluorophore labelled peptide used as substrate in *in vitro* SUMOylation assays. The lysines competent for SUMOylation are in bigger font and in bold face. All subsequent images were obtained by observing FITC fluorescence at 519 nm unless otherwise mentioned. (B) SUMOylation reaction products with peptide shown in (A) and SUMO1/SMO-1 resolved on SDS-PAGE gel. The negative control is lane labelled “no ATP”. Bands of free peptide and peptide conjugated with one, two or multiple (n) SMO-1 are marked. The molecular weight marker positions are indicated. (C) Time course of SUMOylation reaction between peptide shown in (A) and SMO-1. Different time points are indicated. O/N means overnight (~12 hours) incubation of reaction. Band identities and molecular weight marker positions are shown as in (B). (D) SUMOylation reaction using FITC-labelled SMO-1 and in absence of any other peptide substrate. The reaction products and the negative control (without ATP) are resolved and marked. (E) De-conjugation of SUMOylated peptides by SENP2 enzyme. The SUMOylation reaction products shown in (B) were run with or without treatment with SENP2. (F) De-conjugation reaction of SMO-1-YFP construct with SENP2. The SDS-PAGE gel is imaged by observing YFP fluorescence. (G) SUMOylation reaction between FITC-labelled SMO-1 and HMR-1A, a known protein substrate of SMO-1 in *C*. *elegans*. The reaction product band is marked. Negative controls–without ATP and without HMR-1A is also shown. (H) SUMOylation reaction described in (G) is shown with the negative control where all lysines of HMR-1A were mutated to arginines to create the SUMOylation incompetent HMR-1A-K2R construct.

To assess the ability of SMO-1 to be conjugated and deconjugated to substrates we developed an *in vitro* assay. As most of the E1 and E2 interacting residues are conserved or similar in SMO-1, we used the human E1 and human E2 (Ubc9) protein for assays. As a substrate we used a synthetic peptide harbouring two consensus SUMOylation sites found in the IE2 protein (Fig 6A). To facilitate detection of SUMOylated peptide on SDS-PAGE gels we labelled the peptide with Fluorescein iso-thiocyanate (FITC) fluorophore. SMO-1 conjugation to the peptide should significantly change the migration rate of the labelled peptide. Rapid SMO-1 conjugation was observed on the substrate in an *in-vitro* reaction (Fig 6B and S4 Fig), that is comparable with the SUMO1 conjugation. When the conjugation was performed in a time-dependent manner, SMO-1 steadily conjugated to the substrate with time (Fig 6C). Higher molecular bands were observed at longer time points, indicating that SMO-1 can form poly-SMO-1 chains on a substrate. To verify, we carried out an *in-vitro* reaction with FITC tagged SMO-1. Even in the absence of a substrate, poly-SMO-1 chains were observed in solution, indicating that SMO-1 is highly reactive (Fig 6D).

### SUMO proteases can cleave SMO-1 chains

DeSUMOylation enzymes can cleave SUMO chains, making the process reversible to enable another level of control over the SUMOylation process. To examine if the SMO-1 chains can be cleaved by a typical deSUMOylation enzyme, substrate-polySMO-1 conjugates were treated with a human deSUMOylation enzyme, SENP2. Fig 6E shows that SENP2 could efficiently edit the SMO-1 chains from the peptide. To check if the SENP2 can also mature SMO-1, we incubated SMO-1-YFP with SENP2. Fig 6F shows that SENP2 could cleave SMO-1 from SMO-1-YFP, indicating that SMO-1 C-terminal region is homologous to SUMO1/2.

### SMO-1 assembly on the E cadherin homolog HMR-1A

HMR-1, E cadherin homolog, is the only classic cadherin expressing gene in *C*. *elegans*[47,48]. It expresses two isoforms HMR-1A (in epithelial and neuronal cells) and HMR-1B (neuronal cells). HMR-1A is required to form adherens junctions (AJs) which facilitate cell-cell interactions during epidermal morphogenesis in *C*. *elegans* development[29]. HMR-1A could be conjugated with human SUMO1 and the conjugation site lies in the C-terminal cytosolic tail of HMR-1A[29]. To test whether HMR-1A is a substrate for SMO-1 conjugation *in-vitro*, we incubated HMR-1A with the SUMOylation enzymes E1, E2 and SMO-1 in our assays. The SMO-1 protein is labeled with FITC to help in detection. HMR-1A C-tail was found to be SMO-1 conjugated (Fig 6G). HMR-1A C-tail substrate with poly-SMO-1 chain could not be distinguished in the background of unanchored poly-SMO-1 chains. To resolve this we used a mutant (where all the 4 lysines were mutated to arginines) (K2R) construct of C-terminal tail of HMR-1A. The HMR-1A-K2R mutant has similar secondary structure content as the wild-type HMR-1A (S5 Fig). When we repeated the reaction with a HMR-1A C-tail where all possible SUMOylation sites are mutated, HMR-1A-SMO-1 conjugation could not be detected (Fig 6H). These results concur with the earlier report that HMR-1A C-tail is a substrate for SUMO conjugation[29].

## Discussion

SUMO conjugation plays diverse roles in the overall development of *C*. *elegans* including but not limited to germline, pharynx and vulval development[49]. Recent proteomics data have identified new protein substrates of SUMO involved in other signalling pathways and post-translational modifications[49]. Despite its importance, the molecular details of the SUMO homolog in *C*. *elegans*, SMO-1, were unknown. Here, we report the high resolution solution structure of SMO-1, which shows a flexible N-terminal tail, a structured beta-grasp fold and a short-flexible C-terminal tail similar to human SUMOs.

NMR based measurements confirmed that the N-terminal region is highly flexible (Fig 4). In contrast, the long loop between β4 and β5 strand is well ordered and rigid. The importance of the N-terminal dynamic region in SMO-1 is unclear. There is little identity in the N-terminal region between SMO-1 and SUMO1/2 (Fig 1). While SUMO1/2 has lysines in the N-terminal tail which can be SUMOylated to generate poly-SUMO chains there is no such lysine in this region of SMO-1. It was recently observed that the disordered N-terminal region can increase the entropy and reduce the aggregation propensity of SUMO, which helps to keep the protein stable and functional[50]. The N-terminal region of SMO-1 may increase the SMO-1 stability and help to maintain a functional pool of SMO-1 in cells. It will be interesting to understand the exact role of N-terminus tail of SMO-1 in the *C*. *elegans* life cycle.

While higher order organisms like humans express up to five SUMO paralogs, *C*. *elegans* express a single SUMO gene. The human SUMOs exhibit functional heterogeneity between them. For example, SUMO2/3 is known to be expressed in higher amounts than SUMO1 and is conjugated and de-conjugated more rapidly than SUMO1[51,52]. Several SUMO receptors identify one form of SUMO over others[53,54]. It is interesting to postulate how the single gene *SMO-1* is able to combine the functions of the multiple SUMO isoforms. At sequence level, SMO-1 is more identical to SUMO1 (59% identity) than to SUMO2 (47% identity), and hence, it is expected to function similar to SUMO1. However, we find that the electrostatic surface features of SMO-1 resemble more SUMO2/3 than SUMO1 (Fig 3). In particular, SMO-1 has a less positively charged SIM-binding interface (Fig 3). Hence, SMO-1 appears to have combined the features of SUMO1 and SUMO2. Homozygous worms with SMO-1 knockdown have reproductive defects[27,55]. It is not surprising that expression of SUMO1 or SUMO2 can partially rescue the absence of SMO-1 in reproduction[55]. As the single SUMO protein has to be recognised by downstream effector protein of multiple pathways in *C*. *elegans*, there might exist additional levels of regulation to ensure precise cross talk between pathways.

SUMO modification of substrates provide new interactions with receptors via the SUMO:SIM interactions. SMO-1 binds typical SIM like PML-SIM and IE2-SIM with similar affinities (K_d_~ 100 μM) as observed for human SUMO. The binding site in SMO-1 is in the region between β2 and α1 (Fig 5), which is the canonical SIM binding interface. Recent research has indicated that a complex network of SUMO:SIM interactions regulate RING Complex assembly during female meiotic chromosome congression in *C*. *elegans*[56]. Our binding studies indicate that the SUMO:SIM binding mode is probably conserved in *C*. *elegans*. Further studies of the interaction between SMO-1 and SIMs from *C*. *elegans* proteins is required to comprehend the exact mode of interaction in context of the worm development.

SMO-1 is found to be functionally similar to SUMO1/2 in *in-vitro* assays. SMO-1 can form poly-SMO-1 chains on a typical substrate that includes SUMOylation sites (Fig 6). The poly-SMO-1 chains can be depolymerised by a typical human de-SUMOylating enzyme, SENP2. The SMO-1 can also be matured by SENP2, indicating that the C-terminal region of SMO-1 behaves similar to SUMO1/2 and can be identified by SENP2. In addition, SMO-1 could conjugate to its natural substrate HMR-1A (the E-cadherin homolog) *in-vitro*. Ability of SMO-1 to polymerize and form chains may increase the set of functions attributed to SMO-1 and SMO-1 conjugation.

In conclusion, we have characterized the high resolution structural, dynamical and functional features of SMO-1 protein of *C*. *elegans*. It was found that SMO-1 combines the structural and functional features of the different human SUMO proteins, which presumably allows it to function as a single gene in *C*. *elegans*. The data presented here is expected to guide future research in understanding the role of SUMO in development of *C*. *elegans*.

## Supporting information

S1 Fig Interaction surface patch around His75 in SUMO proteins. (A) Structural alignment of SUMO1 (2NIV.pdb) and SMO-1. The chain is shown in ribbon and the residue His75 (in SUMO1) and Asp68 (in SMO-1) are shown in sticks. The electrostatic surface of SUMO1 (B) and SMO-1 (C) is shown in an orientation same as that in (A). Color gradient scheme of the electrostatic surface is shown for each surface. Positively charged surfaces are colored blue, neutral surfaces are in white and negatively charged surfaces are in red. The unit of the color gradient shown is kcal/(mol.*e*). The interaction surface patch around His75 (SUMO1) and Asp68 (SMO-1) is marked by an oval in (B) and (C) respectively.
(TIF)

S2 Fig Fit of peak shifts to binding model. Fit of SMO-1 peak shifts against [PML-SIM]/[SMO-1] ([Lt]/[Pt] in the plots) ratio yielded K_d_ of the PML-SIM/SMO-1 interaction. The titration data was fit in 1:1 protein:ligand model using the equation CSP_obs_ = CSP_max_ {([P]_t_+[L]_t_+K_d_)—[([P]_t_+[L]_t_+K_d_)^2^–4[P]_t_[L]_t_]^1/2^}/2[P]_t_, where [P]_t_ and [L]_t_ are total concentrations of protein and ligand at any titration point.
(TIF)

S3 Fig Interaction of SMO-1 with IE2-SIM. (A) Overlay of the ^15^N-edited HSQC spectra of free SMO-1 (red) with different stoichiometric ratios of IE2-SIM as given in the top left-hand side of the spectra. Three regions of the spectra are expanded to show SMO-1 residue peaks shift upon titration with IE2-SIM. The chemical shift perturbations (CSP) between the free and the bound form were calculated as CSP = [(δ^H^_free_− δ^H^_bound_)^2^+ ((δ^N^_free_− δ^N^_bound_)/5)^2^]^1/2^, where δ^H^ and δ^N^ are the chemical shift of the amide hydrogen and nitrogen, respectively. (B) The CSPs for each residue in SMO-1 upon binding to IE2-SIM. The dashed line indicates twice the standard deviation above average. The residues with CSPs significantly above this line are probably at the interface of the SMO-1/IE2-SIM interaction. The secondary structure alignment of SMO-1 against its sequence is provided above the plot. (C) Modelled structure of SMO-1 (orange) bound to IE2-SIM (dark green). SMO-1 residues which showed CSP higher than twice the standard deviation above average is coloured green.
(TIF)

S4 Fig Raw, unmodified and uncropped, source images of gels shown in Fig 6. The portions of the raw image used for the Fig 6 are marked by rectangular box. Wells marked by “x” are not relevant to this study. (A) Raw gel image of Fig 6B. “SMO-1*” denotes 1:10 ratio of FITC-peptide:SMO-1. “SUMO1*” denotes reaction performed with a different batch of E1 enzyme. (B) Raw gel image corresponding to Fig 6C. (C) Raw gel image for Fig 6D and Fig 6G (the box marked with “*”). (D), (E) and (F) Raw gel image for Fig 6E, Fig 6F and Fig 6H respectively.
(TIF)

S5 Fig Far UV circular dichroism profile for wildtype and K2R mutant of C-tail HMR-1A. (TIF)

## Abbreviations

wt: wild type
SIM: SUMO Interacting Motif
