# Supplementary figures and images for: Structural and functional analysis of SMO-1, the SUMO homolog in Caenorhabditis elegans
Source: PLoS One. 2017 Oct 18;12(10):e0186622. doi: 10.1371/journal.pone.0186622 (PMC5646861; doi:10.1371/journal.pone.0186622)

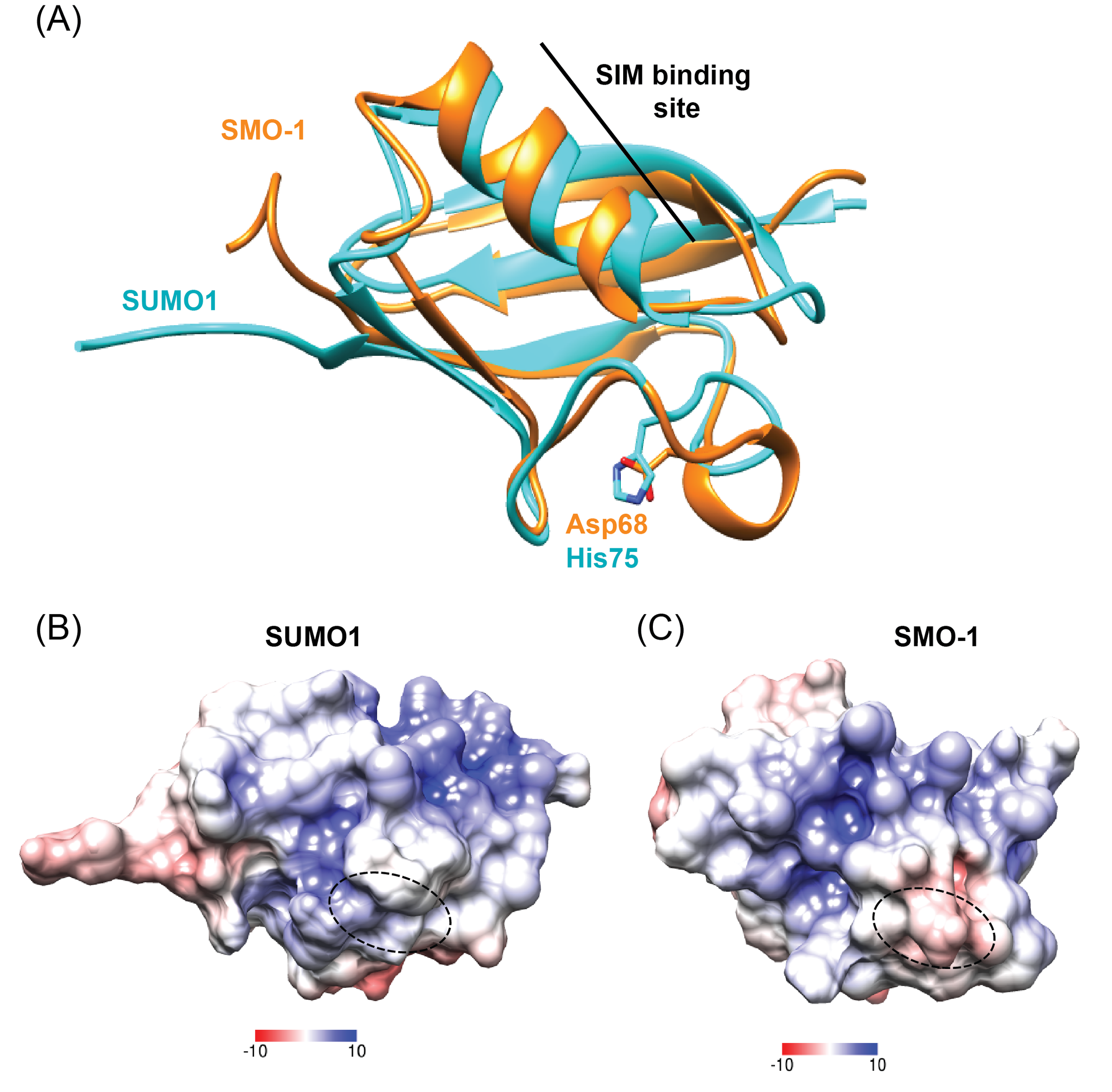

Supplement: S1 Fig — (A) Structural alignment of SUMO1 (2NIV.pdb) and SMO-1. The chain is shown in ribbon and the residue His75 (in SUMO1) and Asp68 (in SMO-1) are shown in sticks. The electrostatic surface of SUMO1 (B) and SMO-1 (C) is shown in an orientation same as that in (A). Color gradient scheme of the electrostatic surface is shown for each surface. Positively charged surfaces are colored blue, neutral surfaces are in white and negatively charged surfaces are in red. The unit of the color gradient shown is kcal/(mol.e). The interaction surface patch around His75 (SUMO1) and Asp68 (SMO-1) is marked by an oval in (B) and (C) respectively. (TIF) [file pone.0186622.s001.tif]

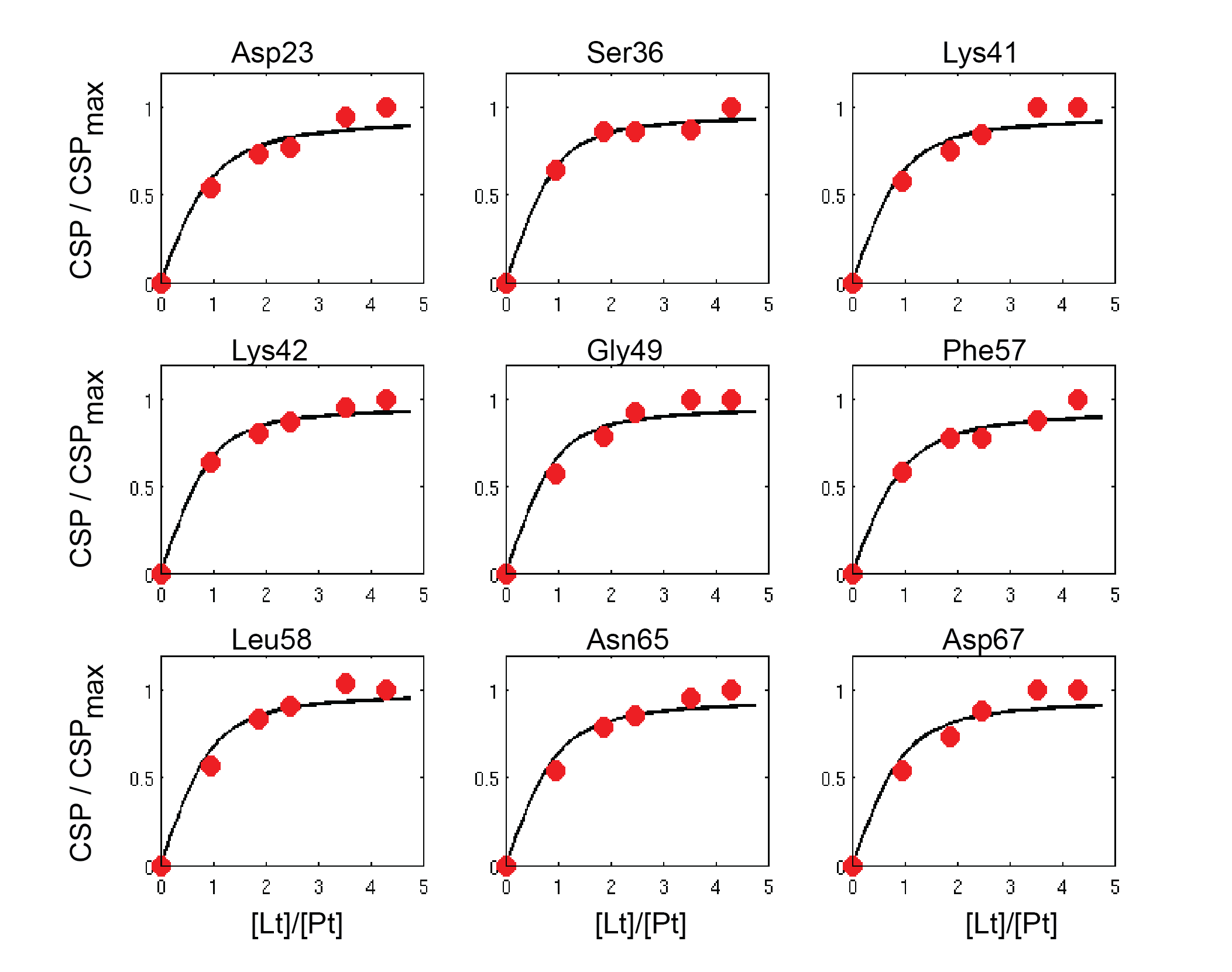

Supplement: S2 Fig — Fit of SMO-1 peak shifts against [PML-SIM]/[SMO-1] ([Lt]/[Pt] in the plots) ratio yielded Kd of the PML-SIM/SMO-1 interaction. The titration data was fit in 1:1 protein:ligand model using the equation CSPobs = CSPmax {([P]t+[L]t+Kd)—[([P]t+[L]t+Kd)2–4[P]t[L]t]1/2}/2[P]t, where [P]t and [L]t are total concentrations of protein and ligand at any titration point. (TIF) [file pone.0186622.s002.tif]

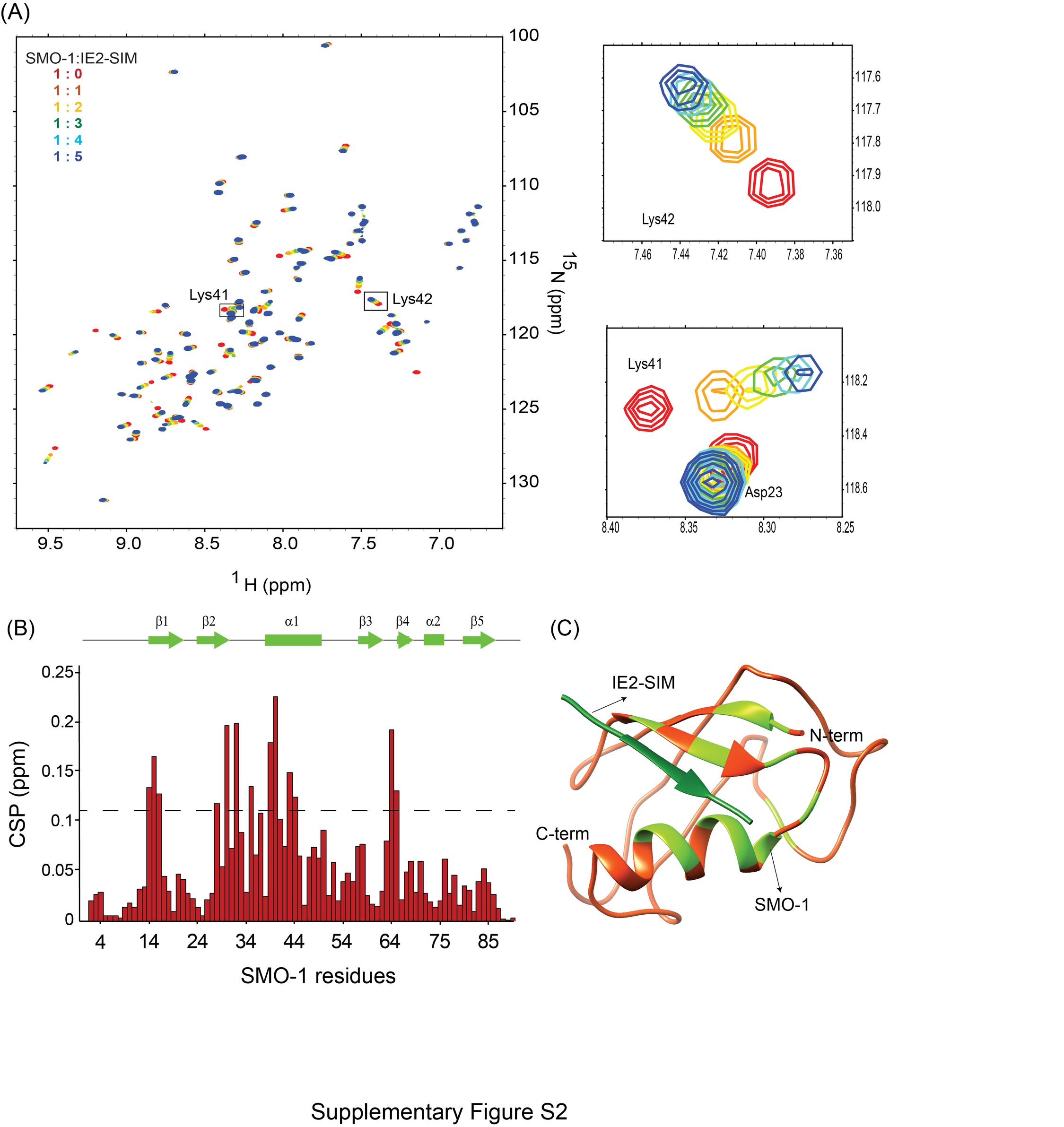

Supplement: S3 Fig — (A) Overlay of the 15N-edited HSQC spectra of free SMO-1 (red) with different stoichiometric ratios of IE2-SIM as given in the top left-hand side of the spectra. Three regions of the spectra are expanded to show SMO-1 residue peaks shift upon titration with IE2-SIM. The chemical shift perturbations (CSP) between the free and the bound form were calculated as CSP = [(δHfree− δHbound)2+ ((δNfree− δNbound)/5)2]1/2, where δH and δN are the chemical shift of the amide hydrogen and nitrogen, respectively. (B) The CSPs for each residue in SMO-1 upon binding to IE2-SIM. The dashed line indicates twice the standard deviation above average. The residues with CSPs significantly above this line are probably at the interface of the SMO-1/IE2-SIM interaction. The secondary structure alignment of SMO-1 against its sequence is provided above the plot. (C) Modelled structure of SMO-1 (orange) bound to IE2-SIM (dark green). SMO-1 residues which showed CSP higher than twice the standard deviation above average is coloured green. (TIF) [file pone.0186622.s003.tif]

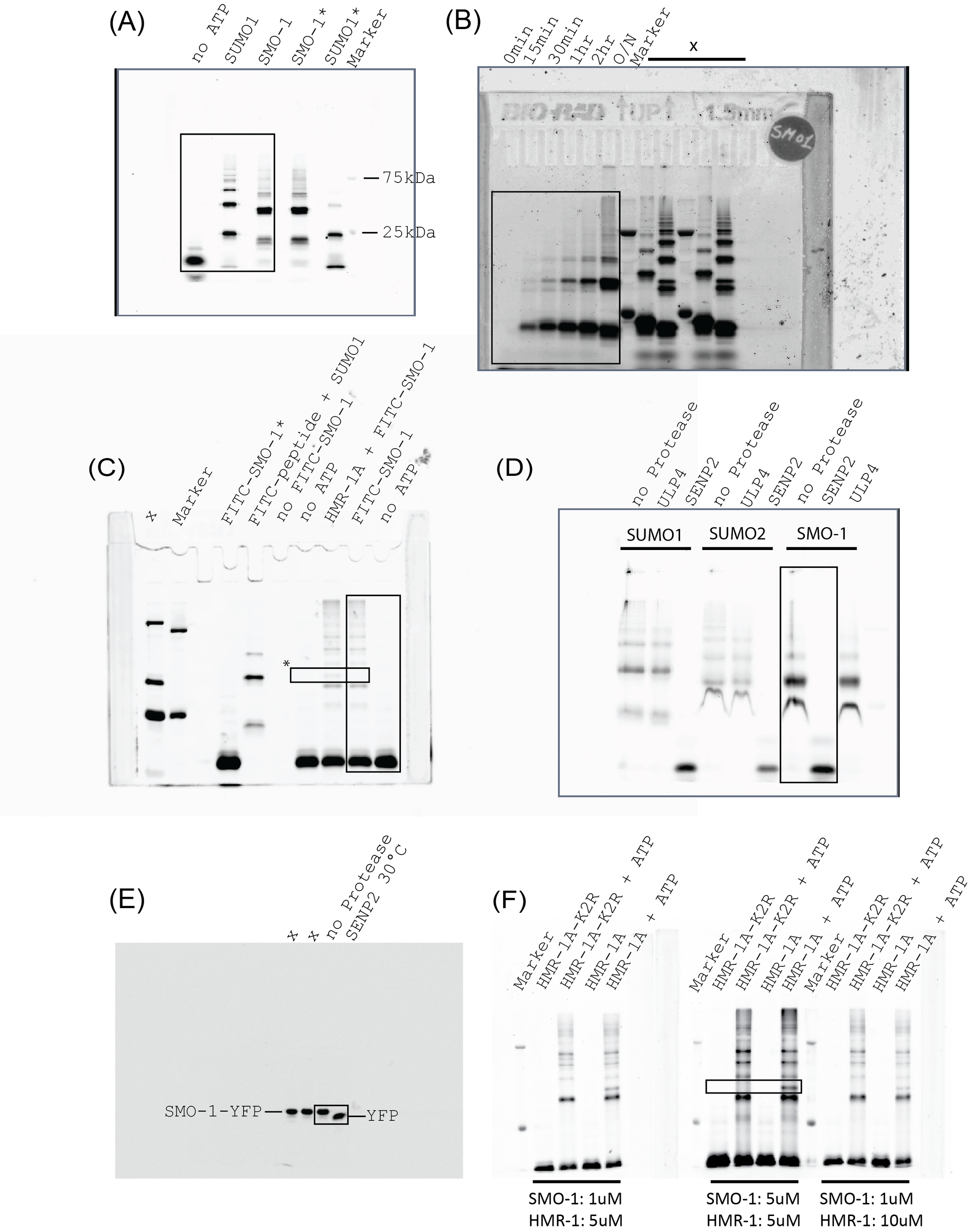

Supplement: S4 Fig — The portions of the raw image used for the Fig 6 are marked by rectangular box. Wells marked by “x” are not relevant to this study. (A) Raw gel image of Fig 6B. “SMO-1*” denotes 1:10 ratio of FITC-peptide:SMO-1. “SUMO1*” denotes reaction performed with a different batch of E1 enzyme. (B) Raw gel image corresponding to Fig 6C. (C) Raw gel image for Fig 6D and Fig 6G (the box marked with “*”). (D), (E) and (F) Raw gel image for Fig 6E, Fig 6F and Fig 6H respectively. (TIF) [file pone.0186622.s004.tif]

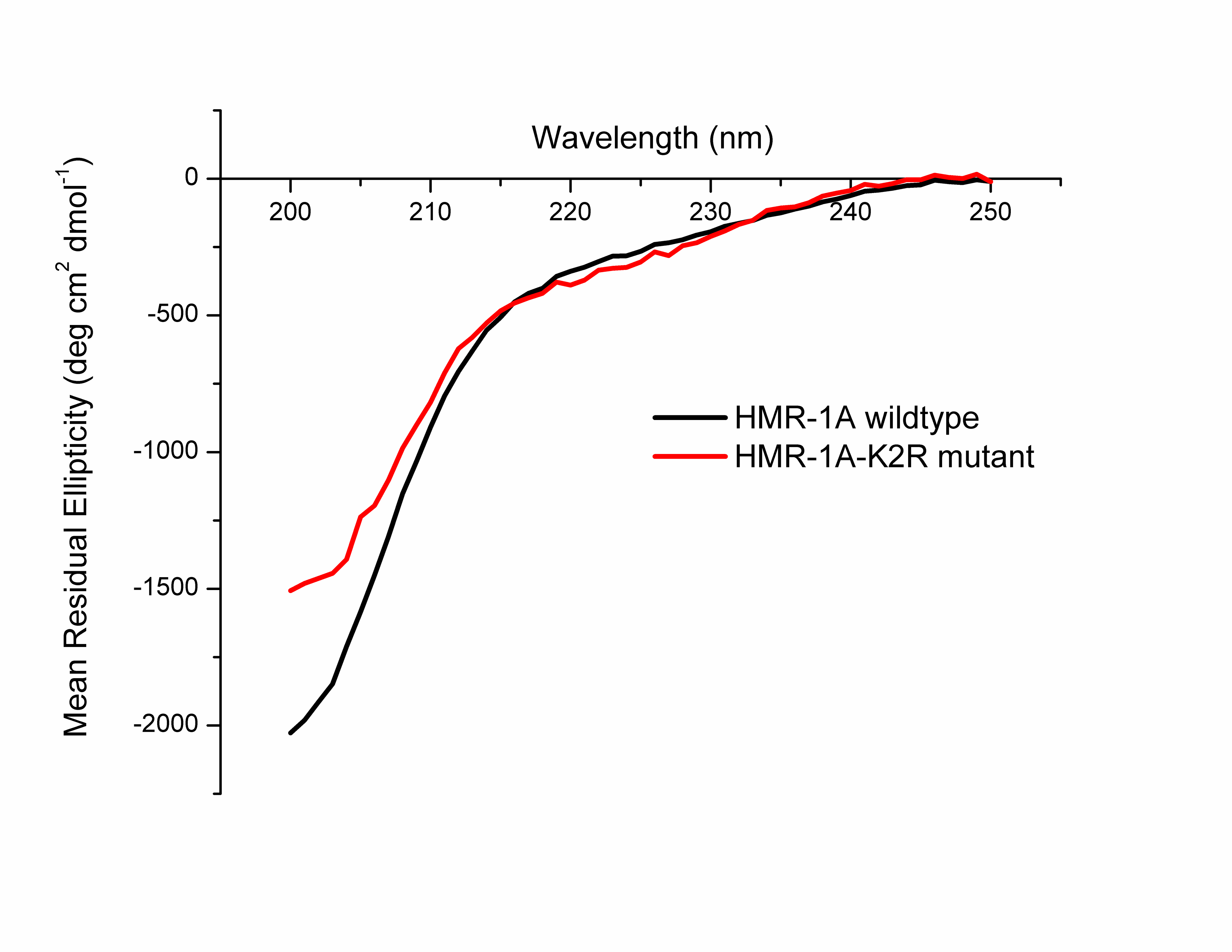

Supplement: S5 Fig — (TIF) [file pone.0186622.s005.tif]
